# Annular pupil confocal Brillouin–Raman microscopy for high spectral resolution multi-information mapping

**DOI:** 10.1515/nanoph-2023-0139

**Published:** 2023-08-25

**Authors:** Yunhao Su, Hanxu Wu, Lirong Qiu, Weiqian Zhao

**Affiliations:** MIIT Key Laboratory of Complex-field Intelligent Exploration, School of Optics and Photonics, Beijing Institute of Technology, Beijing 100081, China; Beijing Institute of Radio Metrology and Measurement (BIRMM), Campus 50 Road Yongding Beijing, Beijing 100854, China

**Keywords:** annular pupil, confocal Brillouin–Raman microscopy, high lateral resolution, spectral broadening suppression

## Abstract

Brillouin–Raman combined confocal spectroscopy is a novel and powerful technique for providing non-contact and direct readout of the micro-regional chemical and mechanical properties of a material, and thus used in a broad range of applications, including material characterization in manufacturing and biological imaging. However, the inadequate spectral and spatial resolution restricts the further development of combined spectral technology. In this paper, an annular pupil confocal Brillouin–Raman microscopy (APCBRM) scheme is proposed to achieve high-spectral-resolution Brillouin spectral detection and high-lateral-resolution Brillouin, Raman, and 3D topography mapping. The use of an annular pupil significantly suppresses the spectral broadening caused by a high-numerical-aperture objective lens and compresses the full width at half maximum of the Brillouin spectrum by 22.1 %, effectively improving the Brillouin spectral resolution. In addition, the size of the excitation spot is compressed, and the lateral resolutions in Brillouin and Raman spectroscopy increased to about 353.2 nm and 347.1 nm, respectively. Thus, high lateral resolution and Brillouin spectral resolution are achieved simultaneously. Furthermore, the high-precision confocal focusing system based on reflected light realizes real-time focusing during scanning and three-dimensional topography mapping. These results demonstrate that APCBRM has excellent potential for applications in the fields of novel materials, precision machining, and biomedicine.

## 1 Introduction

Confocal Brillouin microscopy (CBM) is a non-contact spectroscopic technique for characterizing micro-region mechanical properties such as sample stiffness and viscoelasticity [1, 2]. Confocal Raman microscopy (CRM) is a non-contact and label-free spectroscopic technique used to characterize the micro-region chemical composition and stress distribution of materials [3, 4]. Brillouin and Raman spectroscopy are both inelastic scattering techniques with similar excitation and collection processes, and the chemical and mechanical information in the two spectra are strongly complementary [5]. Therefore, Brillouin–Raman combined confocal spectroscopy has received considerable attention in recent years. In the field of biomedicine, Brillouin–Raman spectroscopy has been used to image the chemical and mechanical properties of cells and tissues at disease lesions to provide novel perspectives for understanding disease development, disease diagnosis, and treatment research [6–9]. In the field of novel materials and precision machining, Brillouin–Raman combined spectroscopy can be used to simultaneously obtain the properties of micro-region components and their crystal form and material elastic moduli *in situ*, which is useful for studying the composite properties of novel nanomaterials such as hydrogel networks and the complex interactions between the material and laser in laser processing [10–14]. Although a significant research upsurge in Brillouin–Raman combined confocal spectroscopy is still maintained to date, several challenges remain to be solved, such as the inadequate spectral and spatial resolution.

Firstly, the Brillouin spectral broadening effect caused by the high numerical aperture (NA) objective severely reduces the Brillouin spectral resolution. The lateral resolution in Brillouin and Raman spectroscopy imaging techniques is limited by the size of the excitation spot in the sample. To reduce the size of the excitation spot and improve the lateral resolution of the imaging system, high-NA objective lenses have been used to achieve submicron lateral resolution at the cost of reduced Brillouin spectral resolution [9, 15, 16]. Because the frequency shift of the Brillouin spectrum is directly related to the angle of excited and scattered wave vectors, the detection result of the Brillouin spectrum is the integral of the contributions from all the scattering angles within the NA range of the objective lens. Therefore, the superposition of Brillouin spectra with different scattering angles over a finite NA broadens the Brillouin spectrum [17–19]. The broadening effect becomes more evident at higher NA and significantly affects the resolution and accuracy of the Brillouin spectrum measurement [20]. To solve this problem, *a priori* model for spectral processing and spatial filters has been used to suppress the spectral broadening effect [17, 18, 21, 22]. However, the calculation process for the former is complicated and cannot be accurately performed for unknown samples, while the latter approach is complex and must be redesigned for objectives with different NAs. In 2014, Battistoni et al. used a spatial filter to select the range of detected phonon vector, thus realizing the suppression of Brillouin spectrum broadening [17]. However, because the spatial filter is placed between the sample and the objective lens, it is limited in the process of spectral mapping. Notably, these methods cannot simultaneously achieve high-lateral-resolution spectral mapping and suppressed Brillouin spectrum broadening.

A second challenge is that the lateral resolution is limited during spectral imaging, especially during surface imaging. Since the lateral resolution improvement that can be achieved by increasing the NA is limited. To surpass the resolution limit, a biaxial optical path and adaptive aberration correction have been used to improve the lateral resolution in Brillouin spectral mapping [23, 24]. The lateral resolution in Raman spectroscopy imaging has been improved by structured light illumination and super-resolution image restoration [25–27]. However, these methods are limited by their complex system structure, poor versatility, and inability to handle both Brillouin and Raman spectroscopy simultaneously.

Finally, existing confocal Brillouin–Raman spectroscopy imaging systems cannot maintain focus with a high accuracy. During the scanning process, the focused spot may become defocused because of sample surface fluctuations, sample stage jitter, or environmental interference, which results in increased spot sizes and poorer imaging quality. It is therefore helpful to maintain spot focusing during spectral imaging to improve the resolution and stability of the system.

To this end, the annular pupil confocal Brillouin–Raman microscopy (APCBRM) technique is proposed in this paper. High lateral resolution and spectral resolution are achieved in APCBRM by using an annular pupil to significantly suppress the Brillouin spectral broadening caused by the high NA of the objective lens. The lateral size of the spot is reduced and the lateral resolution is improved without compromising the spectral detection accuracy. Confocal focusing uses reflected light for high-precision focusing during the imaging process to achieve high-resolution and high-precision chemical and mechanical information imaging.

## 2 Methods

### 2.1 Annular pupil confocal Brillouin–Raman microscopy method

**Figure 1:**
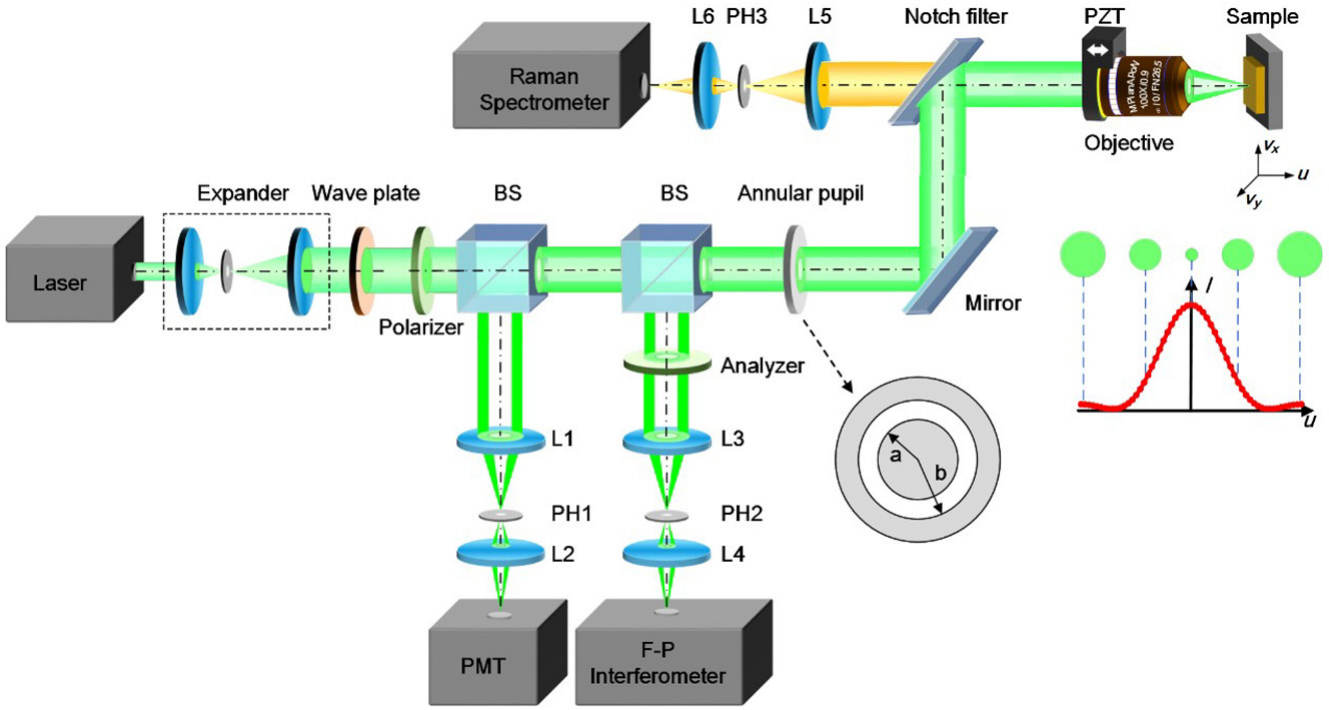
Schematic of the APCBRM system. After beam expansion, the laser reaches the objective through the annular pupil. The light collected by the objective passes through the notch filter to separate the Raman scattering light, and then passes through the lens (L5, L6) and pinhole (PH) into the Raman spectrometer. The reflected and Brillouin scattered light are reflected by the beam splitter (BS) and pass through the lens (L1, L2 and L3, L4) and the pinholes (PH1 and PH2) to reach the photomultiplier tube (PMT) and F–P interferometer, respectively. *a* is the inner diameter of annular pupil, *b* is the outer diameter of annular pupil, and the area between *a* and *b* is the light-passing area. Both the excited light and the scattered light are linearly polarized light with *s* polarization. For cubic crystals and isotropic materials, the Brillouin scattering efficiency is highest at this time.

The principle of APCBRM is shown in Figure 1. A laser passes through a beam expander, half-wave plate, and polarizer between the inner and outer diameters of an annular pupil with the respective sizes of *a* and *b*. The laser light is subsequently focused on the sample by an objective lens to excite Raman and Brillouin scattering lights. The scattered light is collected by the objective and reaches a notch filter (NF). The Raman scattering light passes through the NF and a conjugate pinhole system and is collected by a Raman spectrometer. The Brillouin scattering light and reflected light are reflected by the NF and filtered by the annular pupil. The former is collected by a Fabry–Perot (F–P) interferometer through a polarizer and conjugate pinhole system and the latter by a photomultiplier tube (PMT) through a conjugate pinhole system. The maximum point of the reflected light intensity *I* precisely corresponds to the focus of the APCBRM, thus achieving high-precision axial focusing, ensuring that the focus of each scanning position is focused on the sample surface, and the spot size is the smallest, which can achieve the best spatial resolution of the system.

Compared with traditional confocal spectroscopy microscopy, the key improvement in the proposed method is the introduction of an annular pupil in the illumination and collection paths. The annular pupil modifies the ranges of illumination and collection beams so that the spot size is reduced and the distribution of scattering angles in the system is improved.

A confocal focusing system was constructed based on reflected light from the sample. Our previous study has shown that the confocal focusing system can achieve nanoscale focusing accuracy during spectral imaging. The high accuracy confocal focusing system can suppress the environmental influence in the process of spectral scanning mapping and improve the stability of the system. The height information of the sample can then be collected point-by-point to obtain the three-dimensional morphological information of the scanning area [1, 4, 16]. For the principle and stability verification of the confocal focusing system, see Supporting Information.

### 2.2 Principle of suppressing Brillouin spectrum broadening

**Figure 2:**
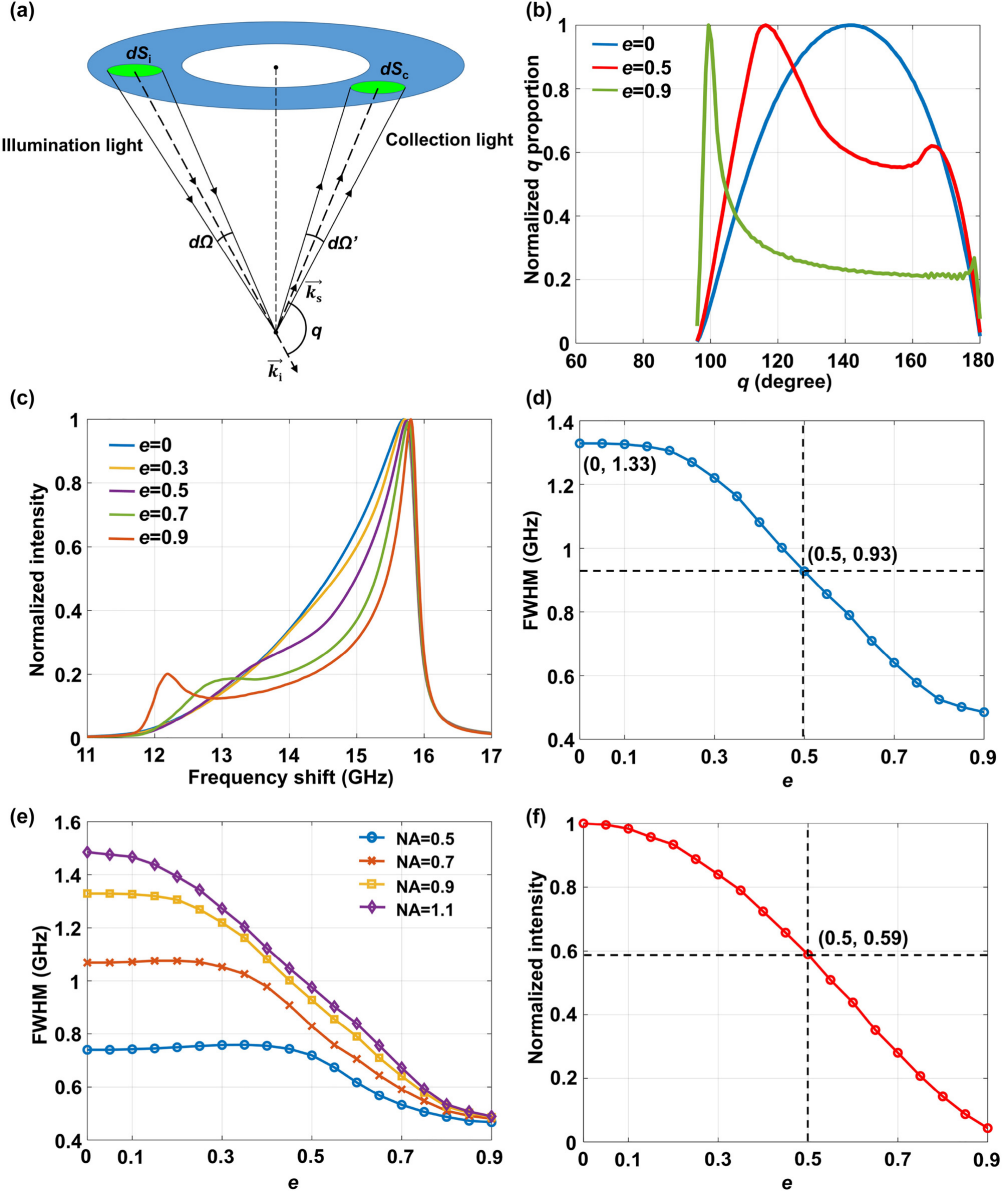
Schematic diagram of Brillouin spectrum broadening compressed by annular pupil. (a) Schematic diagram of scattering angle of annular pupil. (b) The proportion distribution of the scattering angle at different *e*. (c) Simulated Brillouin spectral curves of PMMA at different *e*. (d) FWHM of simulated PMMA Brillouin spectral curve as a function of *e*. (e) FWHM of simulated PMMA Brillouin spectral curve as a function of e under different NA. (f) Curve of the normalized Brillouin spectrum total intensity as a function of *e*.

As shown in Figure 2(a), after adding annular pupil, both the illumination light and the collection light at the center of the objective lens are blocked, and the proportion of the scattering angle *q* of this part decreases correspondingly. The relative proportions of contribution of various scattering angles to Brillouin spectrum during scattering can be changed, and the broadening of Brillouin spectrum can be suppressed, by changing the illumination solid angle range and collection solid angle range of the objective with annular pupil.

Suppose NA = 0.9, Figure 2(b) shows the relative proportion of different scattering angles under different ratio between the inner and outer diameters of the annular pupil (*e* = *a*/*b*). It can be seen that compared to the condition without annular pupil (*e* = 0), the relative proportion of scattering angle in the central range of 120°–170° decreases after adding annular pupil with *e* = 0.5, and the relative proportion of scattering angle in the central range decreases more significantly after adding annular pupil with *e* = 0.9.

The Brillouin frequency shift at a scattering angle is given by [18]

(1) $$v_{B}\left(q\right)\;=\;\frac{2n}{\lambda_{0}}Vsin\left(\frac{q}{2}\right)$$

where *n* is the refractive index of sample; *λ*_0_ is illuminating laser wavelength; *V* is acoustic mode velocity; *q* is scattering angle shown in Figure 2(a).

And the Brillouin spectrum width Δ*v*_*B*_(*q*) for a finite phonon lifetime by

(2) $$\Delta v_{B}\left(q\right)\;=\;\frac{8\pi \eta n^{2}}{\rho \lambda_{0}^{2}}{sin}^{2}\left(\frac{q}{2}\right)$$

where *ρ* and *η* are density and viscosity of sample. The Brillouin spectral distribution at a finite NA can be obtained by integrating the scattering element *s*(*v*, *q*)

(3) $$S\left(v\right)=\underset{\Omega }{\int }\underset{\Omega^{\prime }}{\int }s\left(v,q\right)d\Omega^{\prime }d\Omega$$

over the solid angles of the illumination and collection light paths. The scattering element *s*(*v*, *q*) satisfies the Lorentzian linear distribution

(4) $$s\left(v,q\right)=\frac{{\left(\Delta v_{B}\left(q\right)/2\right)}^{2}}{{\left(v-v_{0}\pm v_{B}\left(q\right)\right)}^{2}+{\left(\Delta v_{B}\left(q\right)/2\right)}^{2}}$$

where *ν*_0_ is the Brillouin frequency shift of the corresponding scattering angle, which can be expressed as:

(5) $$v_{0}=\frac{2n}{\lambda }V\sin\frac{q}{2}$$

Polymethyl methacrylate (PMMA, *η* = 1.2 × 10^−2^ g cm^−1^ s^−1^, *ρ* = 1.19 g cm^−3^, *n* = 1.48, *V* = 2850 m s^−1^) was used as an example, and an excitation wavelength of *λ*_0_ = 532 nm, NA = 0.9 were assumed. The backscattered anti-Stokes spectrum for illumination and collection optical paths with equal NA is shown in Figure 2(c) as a function of *e*. Compared with traditional confocal spectroscopy microscopy (*e* = 0), the narrowing of the Brillouin spectrum curve after adding the annular pupil (*e* > 0) indicates the inhibition of the Brillouin spectrum broadening. As shown in Figure 2(d), the full width at half maximum (FWHM) of the Brillouin spectrum curve decreased with increasing *e*. When *e* = 0.5, the FWHM of the Brillouin spectrum curve decreased by approximately 30.1 % from 1.33 GHz to 0.93 GHz.

NA of objective can affect the effect of annular pupil on Brillouin spectrum broadening. When NA is different, the influence of annular pupil parameter *e* on FWHM is also very different. As shown in Figure 2(e), when NA is low (NA = 0.5, 0.7), the suppressing effect of the annular pupil on broadening is poor, and even aggravates the broadening when *e* is small, which because the annular pupil reduces the spectral contribution of the scattering angle in the central region, Brillouin spectra generated by the scattering angle in the two sides coincide with each other. However, when NA is high (NA = 0.9, 1.1), the Brillouin spectra generated by the scattering angles of the two sides are separated, and the effect is better. And the higher the NA, the larger the *e*, the more obvious the inhibition effect. A dry objective with NA = 0.9 was used in the APCBRM.

However, as *e* increased, the total intensity of the Brillouin spectrum collected by the system also decreased (Figure 2(f)). Suppose NA = 0.9, at *e* = 0.5, the Brillouin spectrum intensity was reduced to 59 % of that of traditional CBM (*e* = 0). Therefore, to achieve a balance between the intensity of the detected spectrum and suppression of the broadening effect at NA = 0.9, a value of *e* = 0.5 was selected for the annular pupil.

### 2.3 Effect of annular pupil on lateral resolution

The 3D intensity distribution function of the APCBRM focused spot can be expressed as

(6) $$\begin{array}{ll} I\left(v,u\right) & ={\left|h_{i}\left(v,u\right)\right|}^{2}\\ & =\left|\overset{\alpha_{0}}{\underset{\alpha }{\int }}P\left(\theta ,u\right)J_{0}\left(\frac{v\;sin\theta }{\sin\alpha_{0}}\right)\exp\left(\frac{iu\sin^{2}\left(\theta /2\right)}{2\sin^{2}\left(\alpha_{0}/2\right)}\right)\right.{\left.\sin\theta d\theta \right|}^{2} \end{array}$$

where *θ* is the beam convergence angle under objective restricted by the annular pupil; (*v*, *u*) are the normalized optical coordinates expressed as *v* = 2π*r*sin*α*_*0*_/*λ* and *u* = 8π*z*sin2(*α*_*0*_/2)/*λ*; sin*α*_*0*_ is the numerical aperture of the objective; *α* is the convergence angle of the objective beam corresponding to the inner diameter of the annular pupil, expressed as sin*α* = *e*·NA.

**Figure 3:**
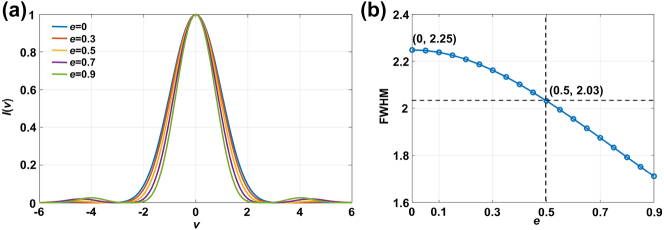
Schematic diagram of annular pupil improving lateral resolution. (a) Lateral intensity curves of focal points at different *e*. (b) FWHM of lateral intensity curve as a function of *e*.

Since the annular pupil reduces the proportion of areas with low NA near the pupil center, the spot size can be compressed laterally [28]. Suppose NA = 0.9, the lateral intensity curves of the focused spots in systems with different *e* were obtained using Eq. (6) and shown in Figure 3(a). Compared with traditional confocal spectroscopy microscopy (*e* = 0), the significantly narrowed FWHM of the lateral intensity curve after adding the annular pupil (*e* > 0) indicates that the lateral resolution of the system is improved. As shown in Figure 3(b), as *e* increases, the FWHM of the lateral intensity curve is also reduced. When *e* = 0.5, the FWHM of the lateral intensity curve decreases by approximately 9.8 % from 2.25 at *e* = 0 to 2.03. The spot size on the sample is therefore compressed, and the spectral lateral resolution of the system improved.

### 2.4 Laser annular pupil confocal Brillouin–Raman microscope

Based on the principle of APCBRM shown in Figure 1, we constructed an annular pupil confocal Brillouin–Raman microscope. A single-longitudinal-mode laser (COHERENT Verdi G2) with a wavelength of 532 nm was used as the excitation source in the APCBRM system together with a 0.9 NA microscope objective (OLYMPUS MPlanFLN 100×). An edge filter (LPD01-532RU-25, Semrock, USA) was used to separate the Brillouin scattering and reflected light from the Raman scattering light, and a high-resolution Czerny–Turner grating spectrometer (FHR1000, Horiba Jobin Yvon, France) was used to collect the Raman spectra. A series multi-pass F–P interferometer (JRS Scientific Instruments TFP-1) was used for Brillouin spectrum acquisition. A piezo motorized two-dimensional translation stage (P-542.2CD, Physik Instrumente, Germany) was used for image scanning and imaging, and a piezoceramic controller-driven objective scanning system (P-725. CD; Physik Instrumente, Germany) was used to drive the objective scanning system. A 10 μm pinhole (Newport, PH-10) and a photomultiplier tube (Hamamatsu, H10723-01) were used to construct the confocal focusing system. The diameters of the pinholes in front of the Raman and F–P interferometers were both 100 μm (Newport, PH-100).

## 3 Results and discussion

### 3.1 Validation of Brillouin spectral broadening suppression

**Figure 4:**
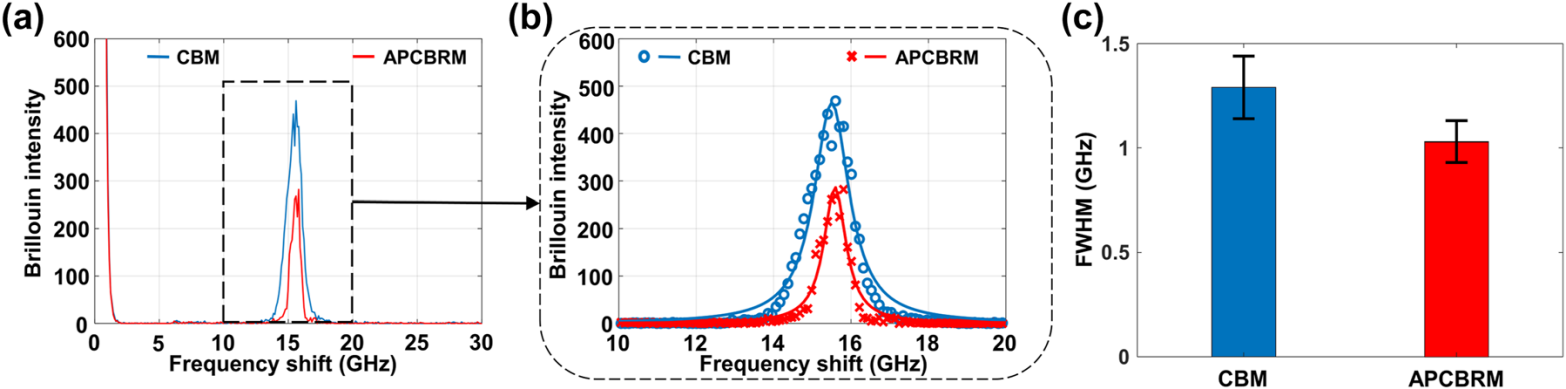
Experimental results of Brillouin spectral broadening suppression. (a) Brillouin spectral curves of PMMA sample probed by CBM and APCBRM. (b) Lorentz fitting results of raw data. (c) Average FWHM and standard deviation of PMMA Brillouin spectral curves obtained using CBM and APCBRM over ten measurements.

PMMA was used as a sample to evaluate the ability of APCBRM to suppress Brillouin spectral broadening. The laser was focused on the surface of PMMA at a power of 50 mW through the objective lens. The time required for the F–P interferometer to acquire a single spectrum was 30 s, and the mirror spacing and scanning range were 2 mm and 300 nm, respectively. The results are shown in Figure 4(a). Lorentz fitting was performed on raw data to obtain the FWHM of the spectral peaks (Figure 4(b)). It can be seen that the Brillouin peak is slightly asymmetrical due to the effect of spectral broadening, which deviates from the ideal Lorentz linear.

The measurements were repeated ten times, and the experimental data fitted using a Lorentz curve. The average FWHM of the Brillouin spectrum collected by CBM was about 1.36 ± 0.13 GHz, while that of the spectrum collected by APCBRM was about 1.06 ± 0.09 GHz, which constitutes a spectral compression of approximately 22.1 % (Figure 4(c)). Therefore, using an annular pupil can significantly suppress the Brillouin spectrum broadening caused by a high-NA objective lens and thus the spectral resolution is improved.

Since the frequency shift and FWHM of the Brillouin spectrum convey mechanical information about the sample, it is necessary to perform fitting on the experimentally obtained spectral data to extract the frequency shift and FWHM information [29]. However, the spectral fitting accuracy is degraded by curve deformation caused by Brillouin spectrum broadening. The measurement accuracy of the Brillouin spectrum can be improved by suppressing this broadening.

### 3.2 Lateral resolution characteristics

**Figure 5:**
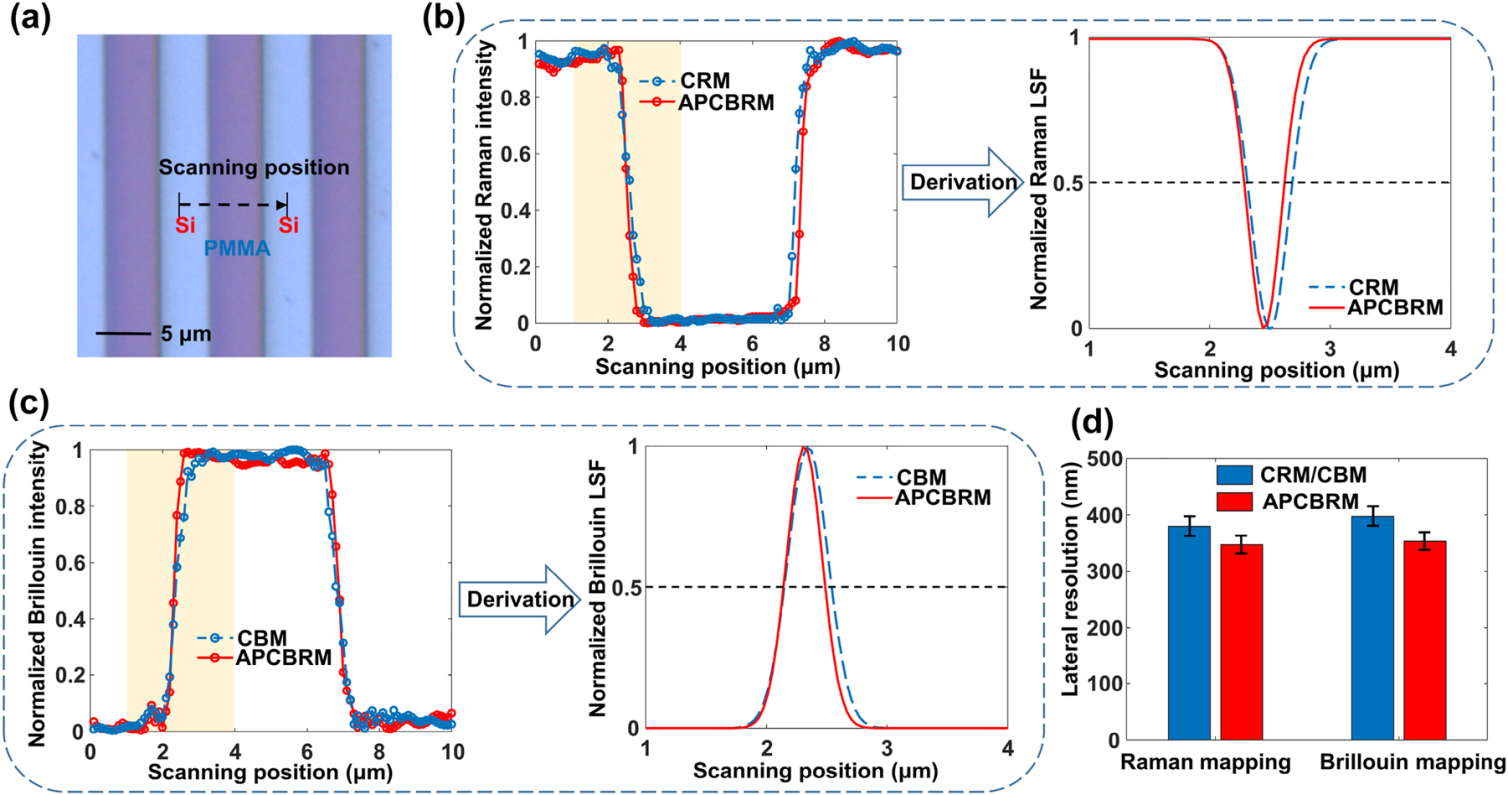
Results of the lateral resolution test. (a) Micrograph and scanning position of the Si-based PMMA striped sample. (b) Normalized Raman intensity curves obtained from traditional CRM and APCBRM scans and normalized traditional CRM and APCBRM Raman LSF curves. (c) Normalized Brillouin intensity curves obtained from traditional CBM and APCBRM scans and normalized traditional CBM and APCBRM Brillouin LSF curves. (d) Mean and standard deviation of traditional CRM/CBM and APCBRM lateral resolutions over ten measurements.

A strip sample (sample height 200 nm) comprising monocrystal silicon (Si) as the substrate and PMMA as the sample pattern material was used to evaluate the lateral resolution of the APCBRM system. Point-by-point Raman and Brillouin scattering signals were collected along the direction perpendicular to the strip, during which the laser spot passed through a Si region, PMMA region, and Si region in turn (Figure 5(a)). During the scanning process, the confocal focusing system was used for real-time focusing to ensure that the focus of the objective lens was always located on the surface of the sample, the spot size was minimal, and the best resolution was achieved in the system. The number of scanning points was 100, the scanning step was 100 nm, and the total scanning distance was 10 μm.

APCBRM with *e* = 0.5 was used for the above scanning process together with CRM and CBM with *e* = 0. The step curves of the normalized Raman intensity as functions of the scanning position are plotted in Figure 5(b) left based on the Si Raman peak near 520 cm^−1^. Notably, the step width of the APCBRM step curve is smaller than that of the CRM curves. The step curves in Figure 5(b) left are taken as the edge spread function curves (ESFs) for the CBM and APCBRM systems. The corresponding linear spread functions (LSFs) shown in Figure 5(b) right are obtained by taking the first derivatives of the ESFs at scanning position between 1 and 4 μm. The FWHM of the curve is taken as the lateral resolution of the system [30]. The step curves of the normalized Brillouin intensity as functions of the scanning position are plotted in Figure 5(c) left based on the Si Brillouin peak near 15.6 GHz. LSFs of CBM and APCRBM are obtained by derivation, and FWHMs of LSFs are obtained.

The measurements were repeated ten times, and obtaining the mean and standard deviation of FWHMs of CRM/CBM and APCBRM LSFs (Figure 5(d)). The Raman lateral resolution of CRM is about 379.8 nm and that of APCBRM 347.1 nm, corresponding to a resolution increase of approximately 8.6 %. The Brillouin lateral resolution of CBM is about 397.4 nm and that of APCBRM 353.2 nm, corresponding to a resolution increase of approximately 11.1 %. These results show that the introduction of the annular pupil resulted in a lateral compression of the excitation spot size and a significant improvement in the lateral resolution of APCBRM compared with that of the traditional confocal spectral imaging system. For analysis and experimental testing of the axial resolution of the system, see Supporting Information.

### 3.3 In-situ topographic imaging, Brillouin mapping, and Raman mapping

Zinc oxide (ZnO) is a direct-bandgap semiconductor material with a wide band gap and excellent photoelectric, mechanical, and chemical properties [31–33]. It is a research hotspot in the micro/nano optoelectronic devices. ZnO micro/nanostructures are typically fabricated through pulsed laser processing [34]. However, the interaction between the pulsed laser and ZnO crystal may cause defects, stress, and changes in the three-dimensional morphology, elastic properties, and thermodynamic parameters of ZnO, which directly affects the performance, reliability, and lifetime of the device [35]. Analyzing the *in situ* morphology and chemical and mechanical properties of processed ZnO can facilitate a better understanding of the fault mechanisms in micro/nano optoelectronic devices for improving the relevant processes.

**Figure 6:**
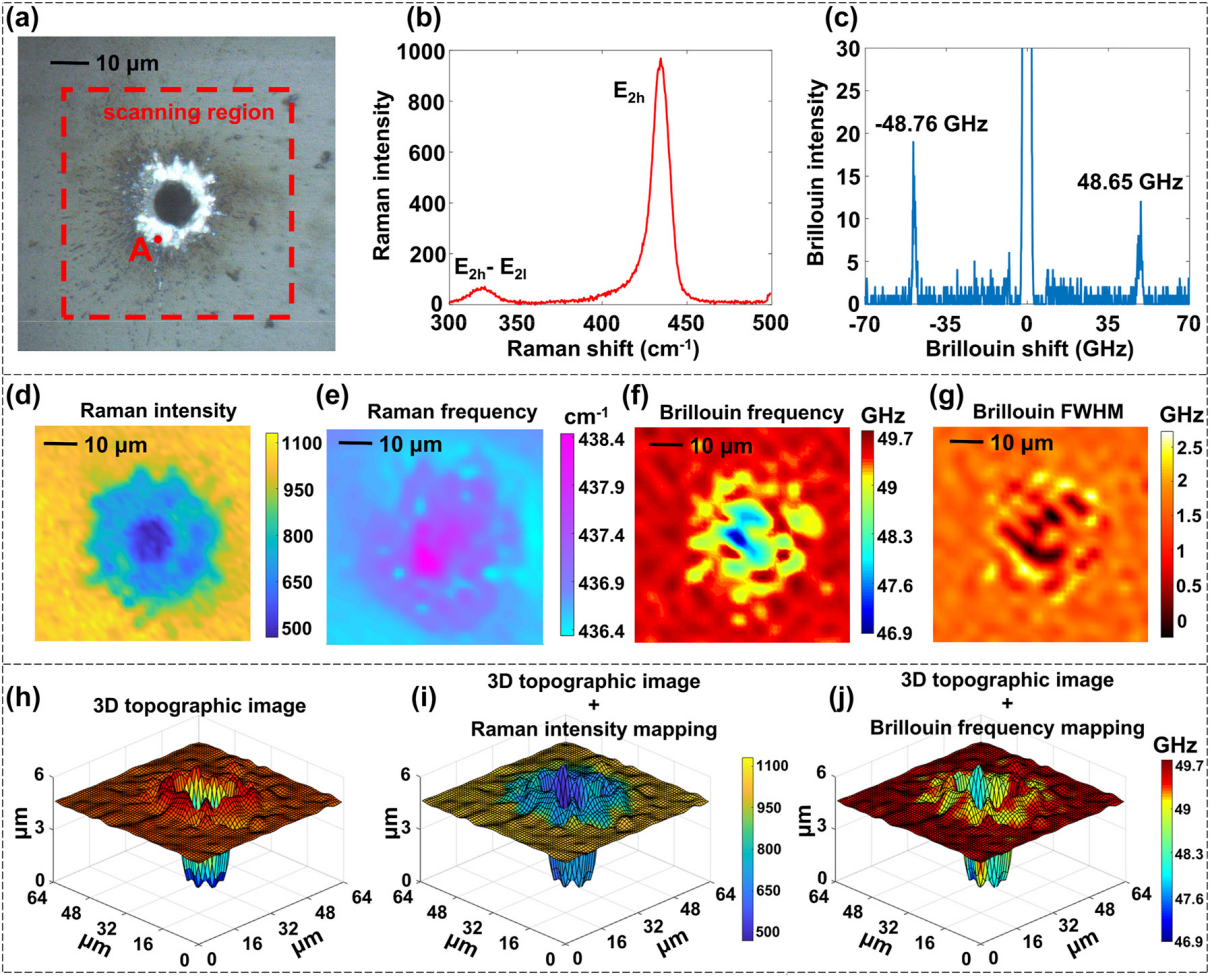
Brillouin-Raman mapping experiments on ZnO samples. (a) Micrograph and scanning region of ZnO sample ablated by nanosecond pulsed laser. (b) Raman spectrum curve of point A. (c) Brillouin spectrum curve of point A. (d) Raman intensity mapping. (e) Raman frequency shift mapping. (f) Brillouin frequency shift mapping. (g) Brillouin FWHM mapping. (h) Three-dimensional topographic image. (i) Fusion image of 3D topographic image and Raman intensity mapping. (j) Fusion image of 3D topographic image and Brillouin frequency shift mapping.

The processed ZnO crystal samples shown in Figure 6(a) were obtained by laser ablation of a ZnO crystal using a nanosecond pulsed laser (Nimma-400, Beamtech Optronics, China). The laser intensity of the sample was approximately 20 mW, and the integration time was 1 s for Raman detection and 30 s for Brillouin detection. The Raman and Brillouin spectral curves at point A are shown in Figure 6(b) and (c), respectively. In the Raman spectrum, the peak near 437 cm^−1^ is the Raman peak corresponding to the signature *E*_2*h*_ vibration mode of ZnO. Its intensity is positively correlated with the regularity of the crystal lattice. The peak near 334 cm^−1^ corresponds to the *E*_2*h*_–*E*_2*l*_ second-order multiphonon mode [36]. The relative change in stress at the scanning position can be characterized through the relative frequency shift of the Raman peak [37, 38]. The relative frequency shifts of the Stokes peak at −48.76 GHz and anti-Stokes peak at 48.65 GHz in the Brillouin spectrum are positively correlated with the elasticity at the scanning position, and the FWHM is positively correlated with the viscosity at the scanning position [29].

The area in the red box in Figure 6(a) was scanned for the imaging experiments using the APCBRM system in a 64 × 64 grid at a scanning step of 1 μm to form a 64 × 64 µm^2^ image. Figure 6(d) shows the spectral intensity mapping of the Raman peak at 437 cm^−1^. The significantly reduced spectral intensity at the laser ablation pit and its surrounding area indicates that the lattice structure of the region was destroyed. Figure 6(e) shows the frequency shift mapping of the 437 cm^−1^ Raman peak. The apparent shift of the spectral peak at the laser ablation pit and its surrounding area to low frequencies indicates that there was stress concentration in this area. Lorentz fitting was performed on the raw measured Brillouin spectra data, and the frequency shift and FWHM of the Stokes and anti-Stokes peaks are obtained by fitting averaged and used to generate Figure 6(f) and (g), respectively. The smaller Brillouin frequency shift at the laser-ablated pit and the surrounding region indicates that the elasticity of these regions was significantly reduced, which may be attributed to the destruction of the crystal structure. The increased Brillouin FWHM in the region near the laser ablation pit indicates an increased viscosity there. However, the FWHM of the laser-ablated pit was approximately zero, possibly because the Brillouin spectrum intensity detected in these regions was too low to fit.

The point-by-point height information can be obtained using the APCBRM system to determine the 3D topography of the scanned area (Figure 6(h)). We fused the Raman intensity mapping (Figure 6(d)) with the 3D topography image to generate the 3D fused image in Figure 6(i). The values on the coordinate axes indicate the 3D topography of the scanned area and the color the distribution of the Raman spectral intensities. Morphological and chemical information on the sample can be simultaneously obtained from the fused image. The Brillouin frequency shift mapping (Figure 6(f)) was fused with the 3D topography image to obtain the 3D fusion image in Figure 6(j), from which morphological and mechanical information on the sample can be simultaneously obtained. These fusion images demonstrate that APCBRM can be used for multi-information imaging of the chemical, stress, mechanical, and three-dimensional morphological information of the sample micro-region, which will greatly facilitate studies on the mechanism, structure design, and performance analysis of micro/nano optoelectronic devices.

## 4 Conclusions

We proposed a novel annular pupil confocal Brillouin–Raman microscopy (APCBRM) for high-resolution geometrical, Raman, and Brillouin spectral imaging. The theoretical analysis and experimental results show that compared with traditional CRM and CBM, the FWHM of the Brillouin spectrum was reduced by approximately 22.1 % in APCBRM, which reflects a significant suppression of the Brillouin spectrum broadening due to a high-NA objective. The Brillouin lateral resolution was increased by about 11.1 %–353.2 nm and the Raman lateral resolution by about 8.6 %–347.1 nm. Therefore, both high lateral resolution and high Brillouin spectral resolution were achieved simultaneously. In addition, the high-precision confocal focusing system based on reflected light can improve the stability of the system and obtain three-dimensional morphological information on the sample. The system was used for mapping experiments on ZnO crystals after pulsed laser processing to obtain *in situ* morphological, chemical, stress distribution, and mechanical information on the laser-processed samples. This demonstrates that the technique is a powerful tool for material science and micro/nano-processing technology.

## Supplementary Material

Supplementary Material Details
