# Supplementary material for: Annular pupil confocal Brillouin–Raman microscopy for high spectral resolution multi-information mapping
Source: Nanophotonics. 2023 Aug 25;12(18):3535–44. doi: 10.1515/nanoph-2023-0139 (PMC11501813; doi:10.1515/nanoph-2023-0139)
Supplement: Supplementary file 1 — Supplementary Material Details [file j_nanoph-2023-0139_suppl_001.pdf]

YUNHAO SU[1], HANXU WU[2], LIRONG QIU[1], WEIQIAN ZHAO[1,*]

Supporting information

1 Axial focusing principle of APCBRM

The process of axial focusing is shown in Fig. S1. PZT drives the objective lens to scan along the axial direction, and the focus position spans the sample surface. The PZT outputs the axial position, the detector behind the pinhole detects the reflected light intensity signal, and the axial intensity response curve is obtained. The axial position of the highest point of the curve corresponds to the location of the focal point on the sample surface. By repeating the above process for each point in the scanning area of the sample surface, the 3D height information of the sample surface can be obtained. At the same time, when collecting spectral signals at each point, the focus is located at the smallest spot on the surface of the sample, which can effectively improve the stability of the system.

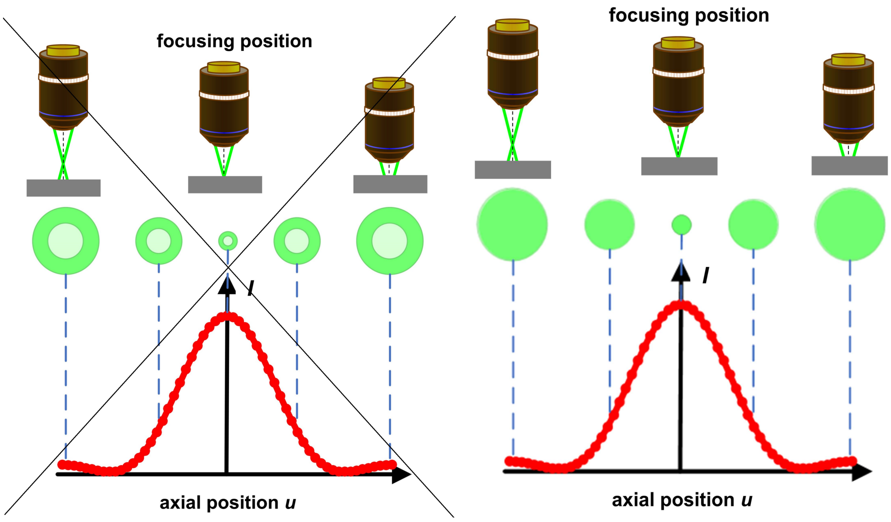

Fig. S1. Axial focusing process

2 APCBRM mapping stability verification

The stability of the APCBRM system was tested by using a strip-shaped sample (the height of the sample is 200 nm) with the substrate material as silicon and the sample pattern material as polymethyl methacrylate (PMMA) as the tested sample. The image size was 80 × 80 pixels, and the scanning step was 300 nm. The CRM\CBM and APCBRM systems were respectively used for spectral scanning, during which the sample was moved down the axis 2μm twice (between P1, P2 and P3). Since the APCBRM system used a confocal focal system to set the focus on the sample surface during each spectrum acquisition (Fig. S2(a)), the axial movement of the sample did not affect the spectral imaging effect, and the fringe pattern was uniform and clear (Fig. S2(b), (c)). However, traditional CRM\CBM system lacks the ability of axial focusing (Fig. S2(d)). After the sample moves in the axial direction, the objective spot defocuses, the spectral imaging effect becomes worse, and the fringe pattern becomes blurred with the increase of the defocusing distance (Fig. S2(e), (f)). Therefore, APCBRM's high-precision axial focusing capability can effectively avoid the influence of axial defocus caused by environmental factors and improve the spectral imaging stability of the system.

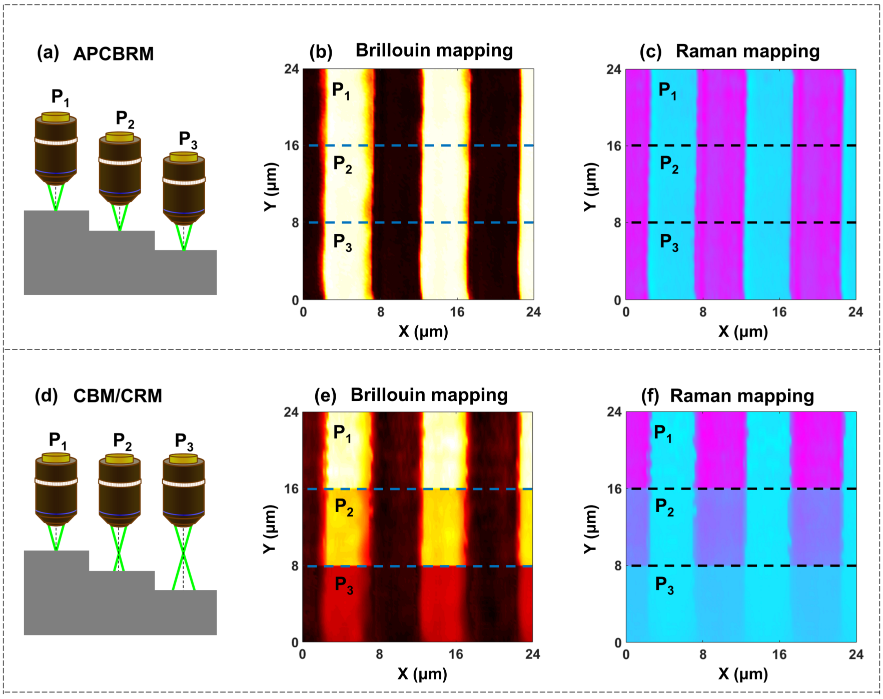

Fig. S2. (a) APCBRM mapping process. The sample was moved axially 2μm twice (between P1, P2 and P3) and the focus remained on the sample surface. (b) The Brillouin spectrum intensity map measured by APCBRM. (c) The Raman spectrum intensity map measured by APCBRM. (d) CRM/CBM mapping process. The sample was moved axially 2μm twice (between P1, P2 and P3) and causing the defocusing. (e) The Brillouin spectrum intensity map measured by CBM. (f) The Raman spectrum intensity map measured by CRM.

3 Axial resolution characteristics

The effect of annular pupil on axial resolution is simulated by Eq. (1).

$$I(v_x,v_y,u)=\left|h_i(v_x,v_y,u)\right|^2=\left|\int_0^{2\pi}\int_a^b P_i(\rho,\theta,u)\exp\left[-i(v_x\rho\cos\theta+v_y\rho\sin\theta)\right]\rho d\rho d\theta\right|^2 . \quad (1)$$

~~(ρ, θ) are the polar coordinates of the pupil function restricted by the annular pupil; (v_x, v_y, u) are the normalized optical coordinates of (x, y, z) expressed as (v_x, v_y) = $2\pi(x, y)\sin\alpha/\lambda$ and $u = 8\pi z\sin2(\alpha/2)/\lambda$; $\sin\alpha$ is the numerical aperture of the objective.~~

$$I(v,u)=\left|h_i(v,u)\right|^2=\left|\int_{\alpha}^{\alpha_0} P(\theta,u)J_0\left(\frac{v\sin\theta}{\sin\alpha_0}\right)\exp\left(\frac{iu\sin^2(\theta/2)}{2\sin^2(\alpha_0/2)}\right)\sin\theta d\theta\right|^2 \quad (6)$$

Where θ is the beam convergence angle under objective restricted by the annular pupil; (v, u) are the normalized optical coordinates expressed as $v = 2\pi r\sin\alpha_0/\lambda$ and $u = 8\pi z\sin2(\alpha_0/2)/\lambda$; $\sin\alpha_0$ is the numerical aperture of the objective; α is the convergence angle of the objective beam corresponding to the inner diameter of the annular pupil, expressed as $\sin\alpha=e\cdot$NA.

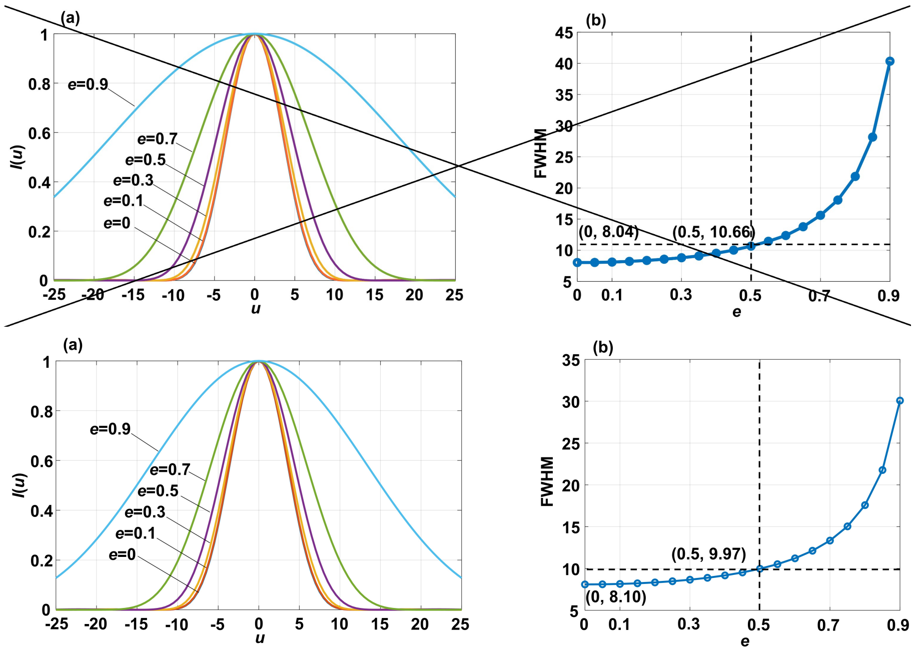

Fig. S3. (a) Axial intensity curves of focal points at different *e*. (b) FWHM of axial intensity curve as a function of *e*.

As can be seen from Fig. S3(a), with the increase of *e*, the FWHM of the axial intensity curve increases, indicating that the axial resolution of the system decreases. As can be seen from Fig. S3(b), when *e*=0.5, the FWHM of the axial intensity curve increases from 8.04 when *e*=0 to 10.66. When *e*>0.5, the FWHM of the axial intensity curve of the system increase rapidly, indicating that the axial resolution decreases rapidly.

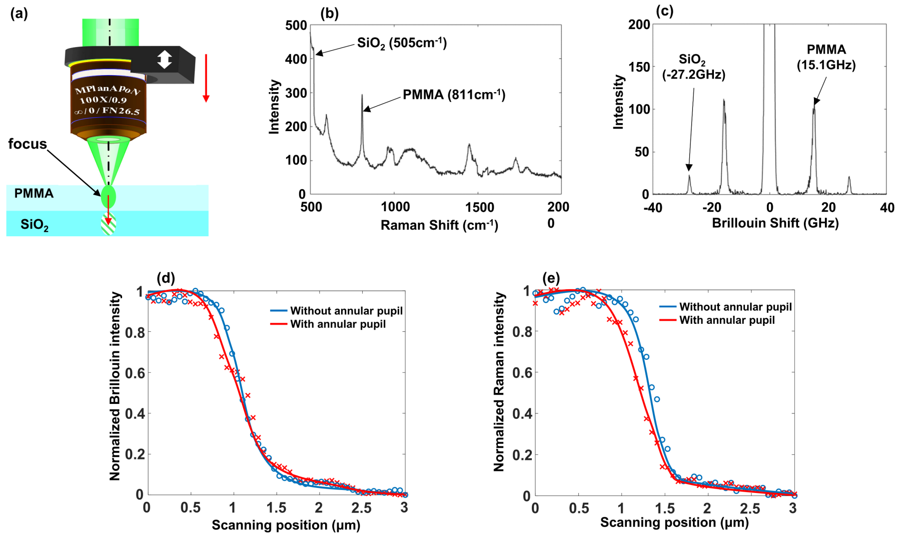

Fig. S4. (a) Schematic diagram of the APCBRM system's axial resolution experiment. (b) The Raman spectrum curve when the spot is located between PMMA and SiO_2. (c) The Brillouin spectrum curve when the spot is located between PMMA and SiO_2. (d) Raman intensity curves of PMMA(811cm^{-1}) with and without annular pupil as a function of scanning position. (e) Brillouin intensity curves of PMMA(15.1GHz) with and without annular pupil as a function of scanning position.

A double-layer transparent sample with the upper layer of PMMA and the lower layer of SiO_2 was used to test the axial resolutions of the system with and without annular pupil. As shown in Fig. S4(a), PZT drives the objective to move the focused spot from the upper layer of PMMA to the lower layer of SiO_2 with the steps of 60 nm, collecting Brillouin and Raman spectrum data of 50 points in total. It can be seen from Fig. S4(d), (e) that after adding annular pupil, the width of the descending edge of the curve of spectral intensity changing with scanning position becomes larger, indicating that the spectral axial resolution decreases.
